# Haloperidol’s Effect on the Expressions of TGFB, NT-3, and BDNF genes in Cultured Rat Microglia

**DOI:** 10.32598/bcn.11.1.1272.1

**Published:** 2020-01-01

**Authors:** Elham Namjoo, Mohammad Shekari, Aliyar Piruozi, Hossein Forouzandeh, Davod Khalafkhany, Abdolvahid Vahedi, Iraj Ahmadi

**Affiliations:** 1. Department of Biology, Faculty of Science, Arsenjan Branch, Islamic Azad University, Fars, Iran.; 2. Genetics and Molecular Biology, School of Medicine, Hormozgan University of Medical Sciences, Bandar Abbas, Iran.; 3. Gerash Cellular and Molecular Research Center, Gerash University of Medical Sciences, Gerash, Iran.; 4. Molecular Biology and Genetics Department, Bogazic University, Istanbul, Turkey.; 5. Department of Physiology, Faculty of Medicine, Ilam University of Medical Sciences, Ilam, Iran.

**Keywords:** Microglia, Haloperidol, TGFB, NT-3, BDNF

## Abstract

**Introduction:**

Microglia, small glial cells, i.e. mesodermal in origin and found in the brain and spinal cord, play a key role in the maintenance of neurons and immune defense. Haloperidol, an antipsychotic drug, is used to treat numerous neurological and neurodegenerative disorders. Its mechanism is not understood; however, haloperidol may result in Wnt signaling pathway activation. This study aimed to activate the Wnt signaling pathway using haloperidol and determining the effect of GSK3 inhibition on the expression of TGFB, NT-3, and BDNF genes in cultured rat microglia.

**Methods:**

Microglia isolation was conducted, and the immunohistochemistry technique was performed to confirm microglia purity. The RNA extraction was followed by cDNA synthesis. Real-time RT-PCR was used to evaluate any significant changes in the expression level of these genes.

**Results:**

The three gene expressions in microglia were proportional to the different concentrations of the drug. More concentration of drugs resulted in higher levels of expression of these genes. Besides, the haloperidol did not affect the expression of the beta-actin gene as the reference gene.

**Conclusion:**

The obtained results supported the beneficial use of haloperidol in targeted microglia therapy. This study can be a breakthrough in neurology research.

## Highlights

- Microglia, small glial cells, play a key role in the maintenance of neurons and immune defense;
- Cells were treated with different doses of haloperidol for 48 h and were accordingly detected under a microscope;
- Using haloperidol to treat diseases with microglia’s involvement in targeted therapy can be effective.

## Plain Language Summary

For microglia isolation, microglia cells were obtained from 4 newborn Wistar rats of 1–2 days age. For the haloperidol treatment, equivalent volumes of cell suspension were transferred to 4 flasks, containing appropriate medium.Microglia isolation was conducted, and the immunohistochemistry technique was performed to confirm microglia purity. The RNA extraction was followed by cDNA synthesis. Real-time RT-PCR was used to evaluate any significant changes in the expression level of these genes. Microglial cells were derived from Wistar rats’ brains and cultured in DMEM medium. The different resistances to trypsin helped microglia purification. An immunohistochemistry assay was performed to verify microglial cells’ purity. Purified cells exposed to specific antibodies were detected bright green, which supported the presence of microglia specific marker, CD163. Finding an approach to stop diseases’ progress or even a stable cure is possible soon. This is due to the last considerable achievements in neuroscience and molecular genetics.

## 1. Introduction

Microglial cells, i.e. among the non-neural cells of the brain, are the unique defense agents of the brain ( Lull & Block, 2010). The origin of microglia has been studied and discussed for years. Studies indicated that microglia arise from progenitors in the embryonic yolk sac, and significantly, appear to persist there into adulthood ( Frick, Williams, & Pittenger, 2013). Approximately 10%–15% of the brain is made up of microglia, highlighting the importance of microglia presence ( Reemst, Noctor, Lucassen, & Hol, 2016). Studies revealed that microglial cells not only function as the first immune sentinels but also have fundamental roles in controlling neuronal proliferation and differentiation ( Ginhoux, Lim, Hoeffel, Low, & Huber, 2015). The number and activity of microglia are strictly controlled. This is because the extra numbers and activity of microglia have the potential to damage the brain tissue ( Graeber & Streit, 2010). Microglia dysfunctions have been identified in several neuropsychiatric conditions; however, it remains unclear whether microglia abnormalities were the cause or the effect of those conditions ( Perry, Nicoll, & Holmes, 2010; Prewitt, Niesman, Kane, & Houlé, 1997). Additionally, the number and function of microglia remain steady under physiological conditions; however, in response to neurodegeneration, microglia multiplies, and adopts an activated state. Microglial cells detect foreign particles, dead cells, and cellular derbies, and swallow them in a healthy brain. Microglial cells, the resident macrophages of the Central Nervous System (CNS), swallow foreign particles and represent foreign antigens on their surface and attract helper T-cells. These cells release cytokines and influence inflammation ( Prewitt et al., 1997, Wohleb, 2016). Identifying microglial cells could be detected in tissue by known microglial markers. There are some established markers for microglial cells and more recently identified markers, including GLUT5, CD163, and CCR2 ( Borda et al., 2008, Graeber and Streit, 2010, Roberts, Masliah, & Fox 2004). WNT/β-catenin signaling is validated as a potent pro-inflammatory regulatory signaling cascade in microglia. WNT signaling, i.e. related to numerous diseases, plays crucial roles in several essential cellular processes, such as cell proliferation, differentiation, migration, and synaptic activity ( Halleskog & Schulte, 2013). Furthermore, 19 human Wnt proteins have been discovered. Wnt signaling inhibits constitutive β-catenin phosphorylation by GSK-3 and allows for β-catenin accumulation, nuclear import, and gene transcription regulation. Defects in this pathway have been linked to many diseases, including Alzheimer’s disease. Studies reported the neuroprotective effects of Wnt pathway activation in neurodegenerative diseases and promoting the differentiation of neural stem cells ( Halleskog & Schulte, 2013; Inestrosa & Varela-Nallar, 2014). The activation of the Wnt signaling pathway can inhibit GSK3, i.e. a growth factor inhibitor. Moreover, to some extent, it affects the expressions of transcription factors of growth proteins, such as TGFB, NT-3, and BDNF ( Tsai, Deng, Lai, Chiu, Yang, & Li, 2014). TGFB proteins are members of a large family, comprised of >30 members in human species. Members of transforming growth factor, beta superfamily, are expressed in different tissues and function at early stages of the development of animals and their lifetime. TGFB pathway plays key roles in regulating cell growth, differentiation, and migration ( Butovsky et al., 2014, Weiss & Attisano, 2013). NT-3 is a member of the neurotrophin family with a critical role in controlling the survival and differentiation of mammalian neurons. The protein encoded by this gene impacts the development of embryonic neurons and is involved in the maintenance of adults’ nervous system ( Joo, Hippenmeyer, & Luo, 2014; Silva-Vargas & Doetsch, 2014). BDNF encodes the brain-derived neurotrophic factor, a neurotrophin family member. BDNF is found in both the central and peripheral nervous system and supports the survival of neurons and results in the growth and differentiation of new neurons and synapses ( Wei, Liao, Qi, Meng, & Pan, 2015). BDNF and NT-3 are members of the neurotrophin family and help to stimulate and control neurogenesis. Studies focused on using NT-3 and BDNF gene therapy to improve central and peripheral nerve functions ( Wei et al., 2015, Weiss et al., 2016). We aimed to investigate the effect of haloperidol on the expression of BDNF, NT-3, and TGFB genes through activating the Wnt signaling pathway.

## 2. Methods

All study procedures were in complete accordance with the National Institutes of Health (NIH) Guide for the Care and Use of Laboratory Animals and were approved by the Ethics Committee of the University. For microglia isolation, microglia cells were obtained from 4 newborn Wistar rats of 1–2 days age. Initially, the study rats were washed using 70% alcohol. Under the sterile condition, the study animals’ brains were removed from the skull and soaked in Hank’s buffer. After removing all extra parts, such as olfactory lobes and capillary networks, the cortex was located in a sterile plate, containing Dulbecco’s Modified Eagle Medium (DMEM) supplemented with 10% FBS and was microdissected using Pasture pipet. Trituration was performed to separate the cells. The isolated cells were transferred to a 50mL flask containing DMEM medium plus 10% FBS. The medium was changed the other day to remove unstuck cells.

For the cellular passage purposes, after 7–8 days, as microglial cells proliferated and grew until 80% confluent, cell passaging was required so that cells receive enough place and nutrients. Accordingly, the medium was removed from the flask, and cells were washed twice with Phosphate-Buffered Saline (PBS). To detach the cells from the flask, an appropriate amount of EDTA-trypsin was added to the flask. After observing separated cells under the microscope, trypsin was blocked by an adequate amount of DMEM medium with 10% FBS. It takes 4–5 hours for cells to fix to the surface again.

The next step was the purification of microglia in the cultured medium. To have pure microglia, EDTA-Trypsin was used. In this method, detecting microglia depends on the different resistance of glial cells to trypsin. Microglial cells are more resistant to trypsin, compared to the other glial cells. Notably, neurons’ sticking to the surface is more time-consuming than that in the microglia cells. Considering these differences, microglia purification was accomplished.

To conduct the immunohistochemistry assay, GFAP, a specific antibody was used to detect CD163 in microglia. The cells were cultured on the coverslips, washed with PBS, and fixed by 4% paraformaldehyde for 15min at 4ºC; then rinsed in PBS and 0.05% Tween 20 for 5min at room temperature. Triton X-100 was used to make cells permeable. After rinsing cells in PBS and 0.05% Tween 20, the cells were incubated in a mixture of goat serum, PBS, and 0.05% Tween 20 for 45min at room temperature. The primary antibody diluted in 0.5% BSA was added to cells, and after incubation at 37ºC for an hour, the cells were washed with PBS and 0.05% Tween 20. The obtained samples were then exposed to the FITC-conjugated secondary IgG antibody and incubated for 20minat 37ºC. Finally, the cells were washed with PBS and 0.05% Tween 20 for 10min in the dark and were observed under a fluorescent microscope.

For the haloperidol treatment, equivalent volumes of cell suspension were transferred to 4 flasks, containing appropriate medium. Next, 2, 4, and 8 mmol of haloperidol solution were added to flasks number 1, 2, and 3, respectively. Flask number 4 was considered as the control. All flasks were placed in the CO2-containing incubator at 37ºC for 48h. Then, a phase-contrast microscope was used to observe the morphology of the cells from all flasks.

The reverse transcriptase (RT)-PCR and quantitative real-time PCR were conducted. After 48h of treating cells with haloperidol and monitoring cells’ morphology, the cells were exposed to trypsin. Then, the medium containing fluent cells was transferred to a 50mL falcon and centrifuged at 2000RPM. The liquid phase was removed, and RNA extraction was performed using the Roche kit according to the manufactures’ instruction. Finally, the RNA pellet was resuspended in 50mL of DEPC-treated RNase-free water. Microtubes containing RNA were incubated for 10min at 58ºC. RNA concentration and purity were assessed by nanodrop1000 spectrophotometer, and 18s and 28s bands were detected on an agarose gel as evidence for RNA integrity. Ferments kit was used for cDNA synthesis. RNA was reverse transcribed with Revert AidTM M-Mulv Reverse transcriptase using random hexamer according to the protocol. Proper primers were designed for three genes and one inner control gene. The cDNA was amplified in a 10 microliter PCR reaction mixture with a specific primer for TGFB, NT-3, BDNF, and B-actin (Table 1). The RT-PCR amplification products of each sample were subjected to ethidium bromide gel electrophoresis, and photographs were captured under UV illumination. Real-time RT-PCR using SYBER Green I fluorescent dye and light cycler was performed according to the instructions to evaluate the genes’ expressions. Pfaffl mathematical model was recruited to calculate the relative expression ratio.

**Table 1.** Primer sequences

**Gene**	**Primer Seq**
TGFB	Forward primer: 5- CCT GGA AAG GGGC TCA ACA C-3
	Reverse primer: 5- CAG TTC TTC GTG GAG CTG A-3
NT-3	Forward primer: 5- AGT GGGCAG CTT TTG CTC-3
	Reverse primer: 5- GTA GAA AGT GGG GGG GAT -3
BDNF	Forward primer: 5- GTACTCTGGAGAGCGTGAATGG-3
	Reverse primer: 5- ACTACTGAGCATCACCCTGGA-3

**Figure 4.**
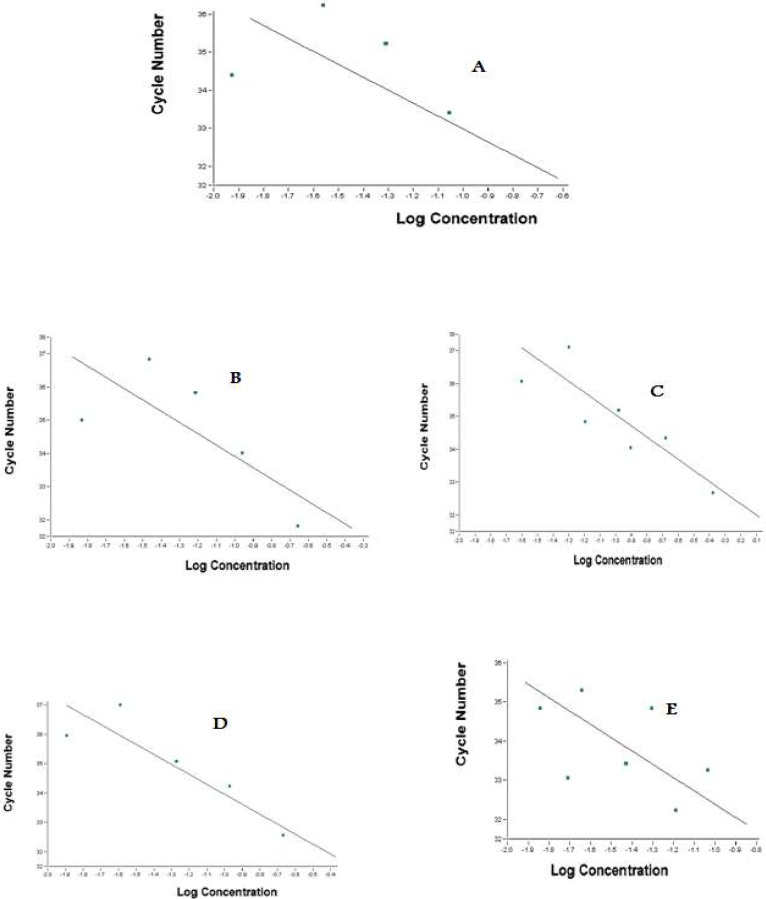
NT-3, BDNF, TGFB, and B-Actin genes’ standard curves A: Standard curve of B-actin gene, reference gene, with a slope of -3.5; B and C: NT-3, and TGFB standard curves with slopes of -3.3 and -3.1; D and E: show the standard curve of BDNF with a slope of -3.3.

Real-time PCR efficiencies were calculated according to the given slopes. Real-time PCR efficiencies rates for TGFB, NT-3, BDNF, and reference genes were 2, 2.08, 2.08, and 1.92, respectively (Figure 4).

**Figure 5.**
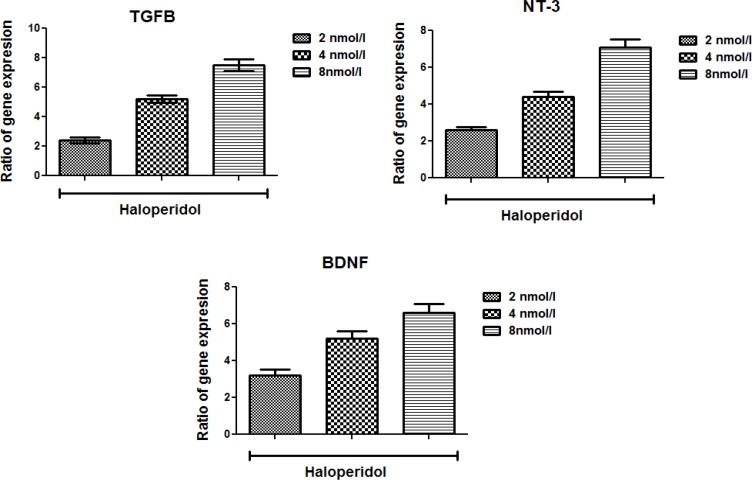
The relative expression rates of TGFB, NT-3 and BDNF in treated microglial cells

The relative quantification of the target genes in comparison to the reference gene was determined using the Pfaffl formula; it calculates the relative expression rates based on the efficiencies and CT of target and reference genes from treated and controlled samples (Pfaffl, 2001). The assay precision was investigated in three repeats within one light cycler run. The obtained data were analyzed by One-way Analysis of Variance (ANOVA). There were significant differences between different dosages’ effects on the expression of the genes (Figure 5).

$$\mathrm{Ratio}=\frac{{\left(E_{\mathrm{target}}\right)}^{\Delta Ct\;\mathrm{target}\left(\mathrm{control}-\mathrm{treated}\right)}}{{\left(E_{\mathrm{ref}}\right)}^{\Delta Ct\;\mathrm{ref}\left(\mathrm{control}-\mathrm{treated}\right)}}$$

## 3. Results

We investigated the effect of haloperidol on the cortex-derived microglia in Wistar rats to identify whether this drug has the potential to activate neurogenesis factors. Microglial cells were grown on a proper medium, and after treatment with haloperidol, the cells’ morphology and genes’ expressions were investigated.

Microglial cells were derived from Wistar rats’ brains and cultured in DMEM medium. The different resistances to trypsin helped microglia purification. An immunohistochemistry assay was performed to verify microglial cells’ purity. Purified cells exposed to specific antibodies were detected bright green, which supported the presence of microglia specific marker, CD163 (Figure 1A, B). The control cells which had not been exposed to the specific antibody demonstrated no color under a fluorescent microscope (Figure 1 C, D). CD163 is a specific marker for microglia; therefore, after purifying microglia through the procedures mentioned above, exposure to the specific antibody visualized microglia under a fluorescent microscope. Microglial cells were observed under a phase-contrast microscope; these small cells had a spindle-shaped nucleus and a few appendages.

**Figure 2.**
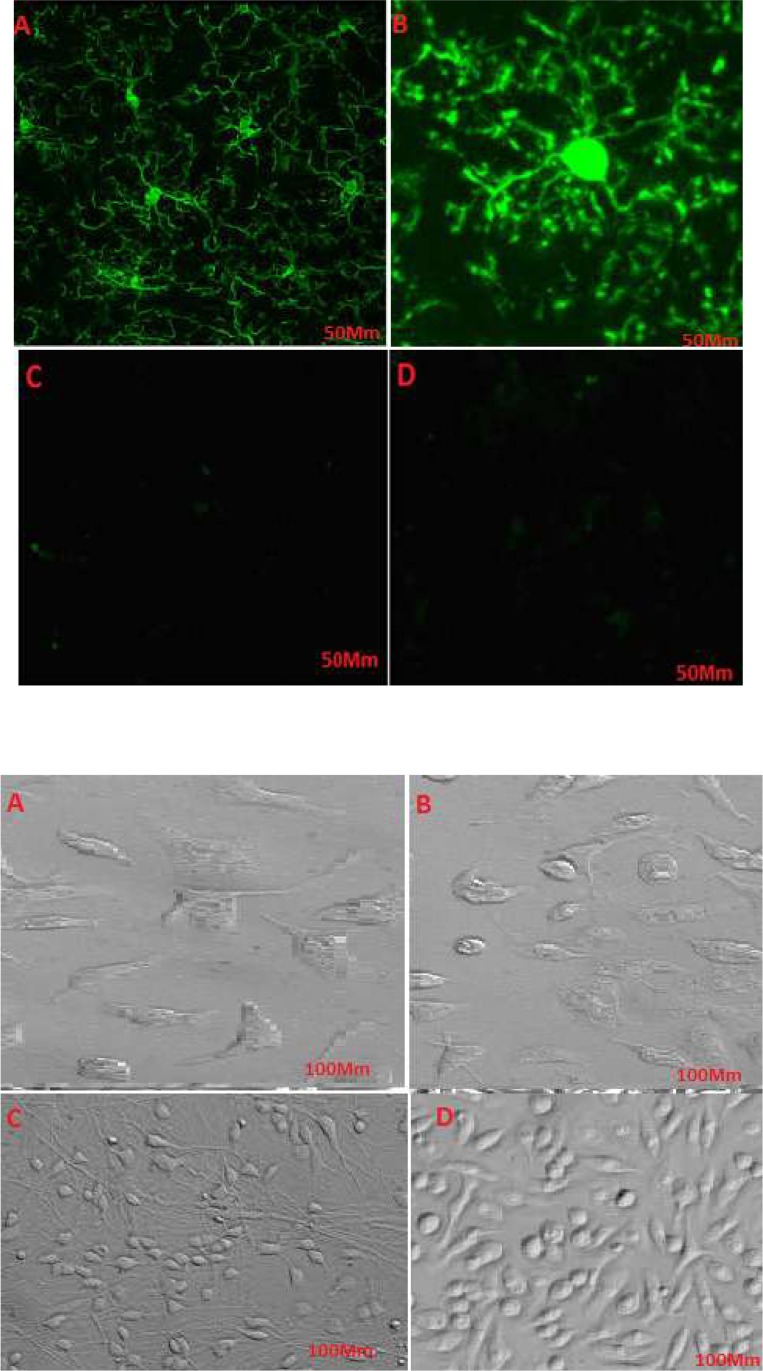
The effects of haloperidol on the morphology of microglial cells A. Control of microglial cells; B. Microglial cells treated with 2 mmol of haloperidol; C. Microglial cells treated with 4mmol of haloperidol; D. Microglial cells treated with 8 mmol of haloperidol A higher dosage of haloperidol causes a more significant increase in the number of cells.

Cells were treated with different doses of haloperidol for 48h and were accordingly detected under a microscope. The number of cells and cells’ appendages significantly increased in the flasks 1, 2, and 3, i.e. treated respectively with 2, 4, and 8 mmol of haloperidol (Figure 2 B, C, D). No significant change was observed in the flask number 4, as the negative control (Figure 2A). The morphological changes in the control cells were negligible compared with the changes in the treated cells. The higher doses of the drug resulted in greater changes in cells’ proliferation. Haloperidol increased the proliferation of cells and the number of appendages in treated cells; thus, it can affect key factors and pathways.

**Figure 3.**
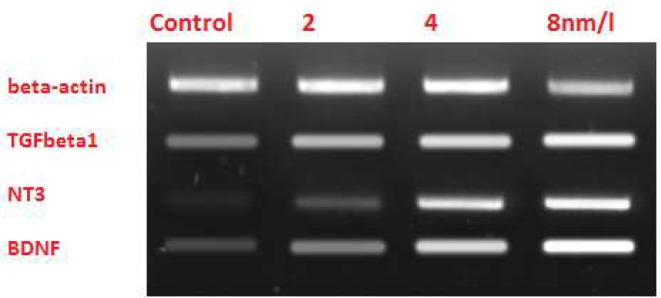
The effect of haloperidol on the expression of neurotrophic factors As it is clear on the gel, higher concentrations of haloperidol resulted in a more considerable change in the expressions of these genes. Expressions of these genes in control cells that had not experienced exposure to haloperidol were unchanged compared to treated cells.

To investigate the effects of haloperidol on microglia’s protective functions, the expressions of TGFB1, BDNF, and NT-3 genes were checked in microglial cells. RTPCR verified the expressions of these genes and specificity of primers; subsequently, the effect of different doses on the expressions of studied genes was visible on the ethidium bromide-stained agarose gel (Figure 3). Then, real-time RT-PCR quantified the alternations of expressions. Alterations in TGFB1, BDNF, and NT-3 expressions were directly proportional to the concentration of haloperidol in each flask. Therefore, higher concentrations of haloperidol resulted in higher expressions of these genes; however, no change was detected in the expression of the B-Actin gene, as the reference gene.

## 4. Discussion

Neurodegenerative diseases are becoming a major health problem; they usually intensify by aging have been the subject of numerous studies ( Korecka, Levy, & Isacson, 2016). Finding an approach to stop diseases’ progress or even a stable cure is possible soon. This is due to the last considerable achievements in neuroscience and molecular genetics. Several studies have supported that most neurodegenerative diseases indicate similar pathological phenotypes ( Skaper, Facci, & Giusti, 2014; Frick, Williams, & Pittenger, 2013; Norden, Muccigrosso, & Godbout, 2015); to some extent, it leads to a common treatment or at least persuades scientist to explore molecular pathways carefully. Numerous studies suggested that microglia play multiple roles in human neurodegenerative diseases. Defects in microglia can impair the performance of some physiological functions, such as derbies phagocytosis or enhance neurotoxic factors’ secretion ( Cartier, Lewis, Zhang, & Rossi, 2014; Von Bernhardi, Eugenín-Von Bernhardi, & Eugenín, 2015).

Furthermore, these impairments are associated with several disorders, such as amyotrophic lateral sclerosis, Alzheimer’s disease, Huntington’s disease, multiple sclerosis, X-linked Adrenoleukodystrophy (X-ALD), and Lysosomal Storage Diseases (LSD). The activated microglia have been implicated in psychiatric disorders, like schizophrenia and mood disorders ( Wolf, Boddeke, & Kettenmann, 2017). Microglia are dynamic cells that gently move through their surrounding microenvironment, like guards, to ensure the absence of imperfections under resting conditions. Through this journey, microglia are primed to detect any injury, homeostatic imbalances, or pathology within the CNS. The presence of each defect results in microglial activation. Microglia are prepared to delay or aggravate neurodegeneration, relying on the balance between the production of trophic versus toxic factors. Aged microglia, like the impaired ones, are not efficient enough to clear derbies and fail in immediately responding to defects ( Monji et al., 2013; Monji, Mizoguchi, & Kato, 2014). Microglia replacement is becoming a critical approach in treating numerous CNS diseases that currently lack efficacious treatments ( Ajami, Bennett, Krieger, Tetzlaff, & Rossi, 2007).

Wnt signaling pathway is essential for microglia to act correctly. Besides, Activating WNT signaling pathway was supported, as a probable treatment for Alzheimer’s disease. Low levels of BDNF, NT-3, and TGFB have been associated with the pathogenesis of several neuropsychiatric and neurodegenerative diseases. Wnt signaling pathway regulates the expressions of BDNF and NT-3 genes and has crosstalk with the TGFB pathway ( Patapoutian, Backus, Kispert, & Reichardt, 1999). Some reports indicated the cooperation of BDNF and WNT signaling ( Hiester, Galati, Salinas, & Jones 2013). BDNF appears to be a direct target of Wnt signaling in glial cells. NTS could regulate the Wnt signaling pathway through the phosphorylation of GSK-3 ( Arevalo & Chao, 2005). There is evidence for the multiple levels of cooperation between the TGFb and WNT signaling pathways in regulating gene expression ( Warner, Greene, & Pisano 2005). Considering the importance of microglia in the maintenance of the brain environment ( Pierre, Smith, Londono, Chemtob, Mallard, & Lodygensky 2017) and the role of Wnt signaling in the appropriate function of microglia, and the key role of neurotrophic factors in neurogenesis, the necessity of exploring these pathways in the brain is accentuated. Based on these consecutive findings, investigating the effect of a recognized drug that influences the Wnt signaling pathway was highly recommended. Haloperidol is a well-known antipsychotic drug with a critical role in treating patients with known psychosis or other behavioral problems. There are numerous studies investigating haloperidol effects on neurodegenerative diseases ( Keilhoff, Grecksch, Bernstein, Roskoden, & Becker, 2010, Shin and Song, 2014).

According to the previous studies, DVL is an important transducer of WNT signaling for the canonical and PCP pathway (Qu). Studies revealed that DVL is activated by haloperidol, i.e. sufficient to activate Wnt signaling pathway ( Sutton, Honardoust, Mouyal, Rajakumar, & Rushlow 2007); therefore, haloperidol could play a key role in activating NTs and TGFB. The present study focused on investigating the effects of haloperidol on the expressions of NTs and TGFB in rat; cultured microglia verified our hypothesis that this drug could increase these genes expression. Changes in the number of microglial cells and their appendages were visible under a microscope, i.e. a good indicator of the direct effect of haloperidol on the proliferation of microglia. Further comprehensive studies are required to explore different effects of this drug on glial cells to clarify if haloperidol can be used as a safe treatment in these disorders or not. BDNF, brain-derived neurotrophic factor, NT-3 are the key factors of neurogenesis ( Pandya, Kutiyanawalla, & Pillai, 2013). Moreover, TGFB revealed a significant increase in their levels of expression. The presence and function of these factors seem to be a chance to heal the wounds in neuropsychiatric and neurodegenerative diseases. The present study fulfilled our aim to clarify the effect of haloperidol on microglial cells and neurotrophic factors’ expression. The present study data are limited to in vitro conditions and not surprisingly different result will be obtained from in vivo experiments due to the interactions of cells with each other, effects of the drug on other glia cells, and the possible effects of surrounding microenvironment on the cells and gene expression. A study reported decrease in the expression level of BDNF in the occipital and frontal cortex of rats after receiving haloperidol treatment ( Angelucci, Mathé, & Aloe, 2000).

## 5. Conclusion

According to our findings, i.e. consistent with previous studies, it is not exaggerating to postulate the accomplishment of this study, as a significant step in neuroscience studies. Using haloperidol to treat diseases with microglia’s involvement in targeted therapy can be effective. To find out more in this field and to be more satisfied with our findings, we suggest future precise and comprehensive investigation, including in vivo experiments.
